# Opportunity costs of attending surgical clinic appointments and experiences with telemedicine for follow-up care

**DOI:** 10.1177/20503121211045247

**Published:** 2021-09-11

**Authors:** Mitchell D Thatcher, Michael W Thatcher, Mckinley C Smith, Michelle McCarron, Jeremy Reed

**Affiliations:** 1 College of Medicine, University of Saskatchewan, Saskatoon, SK, Canada; 2 Research Department, Saskatchewan Health Authority, Regina, SK, Canada; 3 Department of Orthopedic Surgery, University of Saskatchewan, Saskatoon, SK, Canada

**Keywords:** Telemedicine, surgery, opportunity costs, patient satisfaction, orthopedics

## Abstract

**Objectives:**

Telemedicine has been rapidly implemented in orthopedics during the coronavirus (COVID-19) pandemic. The purpose of this study was to quantify opportunity costs for patients attending typical in-person appointments and understand their perceptions of telemedicine for follow-up care.

**Methods:**

A cross-sectional study was performed by surveying patients who had elective orthopedic surgery and attended at least one in-person and one phone call appointment. The survey assessed opportunity costs associated with in-person appointments, experience with telemedicine, and preferred type of future appointment.

**Results:**

Of the 49 eligible patients, 41 (83.7%) completed the survey. The median travel distance to the clinic was 108 km, and the time spent in the clinic was 60 min. Participants responded “yes” to various forms of opportunity costs associated with attending in-person appointments, including missed work (46.3%), lost income (34.1%), recreational activities (26.8%), home or yard care (14.6%), socializing with friends or family (12.2%), school (2.4%), and childcare (2.4%). In addition, elements of the telemedicine appointment were rated from 1 (least favorable) to 10 (most favorable), and averages were calculated for ease of use (9.2), convenience (8.4), confidence in the doctor’s diagnostic ability (8.2), likelihood of using the service again (6.4), and overall satisfaction (8.2). Preferred future appointment types included having the first visit in-person and subsequent visits via telephone (61.0%), in-person only (36.6%), and unsure (2.4%).

**Conclusion:**

This study identifies various opportunity costs associated with in-person orthopedic appointments and a favorable view toward telemedicine for follow-up care.

## Introduction

In March of 2020, the World Health Organization (WHO) declared the coronavirus disease 2019 (COVID-19) a global pandemic.^1^ Telemedicine has been rapidly implemented in outpatient settings to help minimize the spread of the coronavirus,^2,3^ although relatively little is known about its impact on patient productivity and how patients perceive these services. Furthermore, the shift toward telemedicine has provided a chance to reflect on the opportunity costs that patients face when attending typical in-person appointments.

Orthopedic in-person clinic appointments are associated with a variety of opportunity costs for patients. These costs include the time spent traveling to the clinic,^4^ costs related to travel,^5^ and income lost due to missed work.^6^ Furthermore, these losses may be increased for patients who live in rural areas. Researchers have found that for patients receiving carpal tunnel release surgery in Saskatchewan, travel-related expenses such as food, transportation, and lodging are more than $2000 CAD higher for rural patients than urban patients.^7^ Telemedicine may reduce these opportunity costs, and researchers have identified high patient satisfaction scores for both telephone and video calls for outpatient orthopedic visits.^5,8^ The high satisfaction ratings with telemedicine appointments during the COVID-19 pandemic, in particular, are thought to be due to a decreased risk of contracting the infection, reduced travel time, and reduced waiting time to see the doctor.^5,8^ Furthermore, researchers have found that patients actually prefer telemedicine over in-person appointments in some instances.^6^ In a study by Sethuraman et al.,^6^ 45% of post-operative total joint arthroplasty patients preferred not to visit the surgeon’s clinic in-person due to the extensive time required and lost wages.

Regardless of telemedicine’s potential benefits, the service has been vital for continuing to see orthopedic patients during the global COVID-19 pandemic.^9^ Given the potential longevity of the pandemic and for overall healthcare quality improvement, it is important to further explore telemedicine as a method of providing orthopedic care. During the pandemic, the orthopedic clinic of the senior author of this study saw a massive increase in telemedicine utilization, prompting the development of a survey for patients to share their experiences with the new service. Therefore, to build upon previous work in telemedicine, the purpose of this study is to quantify the opportunity costs for patients attending in-person appointments and understand their perceptions of telemedicine for orthopedic follow-up care.

## Methods

### Study design

A cross-sectional study was performed using a telephone survey of patients who had elective orthopedic surgery between 1 July 2019 and 1 July 2020, and a telemedicine appointment between March and July of 2020. The patients were all under the care of a single orthopedic surgeon. The survey was developed according to guidelines on the use of telemedicine research surveys,^10^ and questions were modified to be suitable for orthopedic patients.

The telephone survey was delivered in three sections: (1) opportunity costs faced with in-person clinic appointments, (2) experience with a telemedicine follow-up appointment, and (3) preferred type of appointment for future care. In the first section, participants were asked which city or town they usually travel from (used to calculate median distance) and the estimated time spent in the clinic (including wait time). Participants were also asked if they missed any commitments because of the appointment (including work, education, childcare, recreational activities, home or yard maintenance, and socializing with friends or family) and about any income losses because of the appointment. The second section asked participants to reflect on their telephone appointments with the surgeon. Participants provided a rating on a scale of 1–10 (with 10 being the most positive) of the convenience of telemedicine, ease of use, confidence in the doctor’s ability to diagnose or understand health concerns over the phone, the likelihood of using telemedicine in the future, and overall satisfaction. The survey’s final section assessed the participants’ preferred type of follow-up appointment if they were to have another surgery. Participants were asked to choose between exclusively in-person visits, having the first visit in-person and the rest over the phone, all appointments exclusively by telephone, or to indicate that they were unsure.

### Statistical analyses

Descriptive statistics were used to quantify the various opportunity costs, ratings of different telemedicine elements, and preference for future appointment types. Distance traveled, time spent traveling, and estimated time spent in the clinic were reported as medians with interquartile ranges (IQRs). Missed commitments and categorical data about the amount of income lost due to the appointment were reported as frequencies. Ratings of different elements of the telemedicine appointment were reported as mean values and standard deviations. Preferences for future appointment types were reported as frequencies. The Spearman rank correlation coefficient was used to examine whether (1) distance traveled or (2) time spent at the clinic were related to ratings of the telemedicine appointment’s five elements. Two-tailed, paired-samples *t*-tests were used to determine whether there were differences in ratings for the five telemedicine elements based on preferred future appointment type (in-person only or blended in-person and telephone). When Levene’s test for equality of variances was violated, results are reported with equal variances not assumed. Mann–Whitney *U* tests were used to assess whether either (1) distance traveled or (2) time spent at the clinic were related to a preferred type of appointment for future care.

### Ethics

Ethical approval for this study was obtained from the Institutional Review Board. The need for informed written consent was waived by the Review Board, and approval was granted for informed verbal consent prior to data collection. This study was completed in accordance with the World Medical Association Declaration of Helsinki.

### Inclusion and exclusion criteria

An initial chart review was performed to assess the eligibility of patients for later participation in the telephone survey. Patients were included if they met all of the following criteria: were between 18 and 100 years old, had attended at least one in-person clinic appointment, and attended at least one telemedicine follow-up appointment after their surgery. These criteria were set so that patients could compare their experience with both in-person and telemedicine appointments. Patients were excluded if they had non-elective surgery. No additional exclusion criteria were created. A formal sample size calculation was not performed as all eligible patients identified during the chart review were included for participation.

## Results

### Baseline characteristics

The survey was administered through a telephone call in July of 2020, and research assistants attempted to contact each patient at least three times over 2 weeks. If patients were interested in participating in the study, they were read a consent form outlining the research project, were given the opportunity to ask questions, and were then asked for informed verbal consent. Initial chart review identified 49 patients who were eligible for participation, and 41 (83.7%) completed the telephone survey. Six patients could not be reached after at least three tries, and two declined to participate.

### Study outcomes

Several opportunity costs were identified for attending in-person orthopedic clinic appointments. First, participants reported a median travel distance of 108 (IQR = 17–201) km one-way to attend their appointment and a median amount of time spent in the clinic of 60 (IQR = 30–60) min. Participants were asked to answer yes or no to various forms of missed commitments due to their in-person appointment. These missed commitments are represented in Table 1, and notable findings include the number of participants who typically miss work due to their appointment (46%), lose income (34%), and forgo participating in their usual recreational activities (27%).

**Table 1.** Types of productivity losses associated with attending an in-person orthopedic appointment (*n* = 41).

Type of productivity loss	Frequency of responses (#)	Percentage (%)
Childcare	1	2.4
School	1	2.4
Socializing with friends or family	5	12.2
Home or yard maintenance	6	14.6
Recreational activities	11	26.8
Income	14	34.1
Work	19	46.3

Participants were asked to evaluate their phone call appointment by ranking five elements of the call from 1 to 10 (1 = least positive and 10 = most positive). Participants rated the convenience of the phone call appointment (mean (M) = 8.4 and standard deviation (SD) = 1.8), ease of use (M = 9.2 and SD = 1.2), confidence in the doctor’s ability to understand or diagnose health concerns over the phone (M = 8.2 and SD = 1.9), likelihood of using phone call services in the future (M = 6.4 and SD = 3.0), and overall satisfaction (M = 8.2 and SD = 2.1).

**Figure 1.**
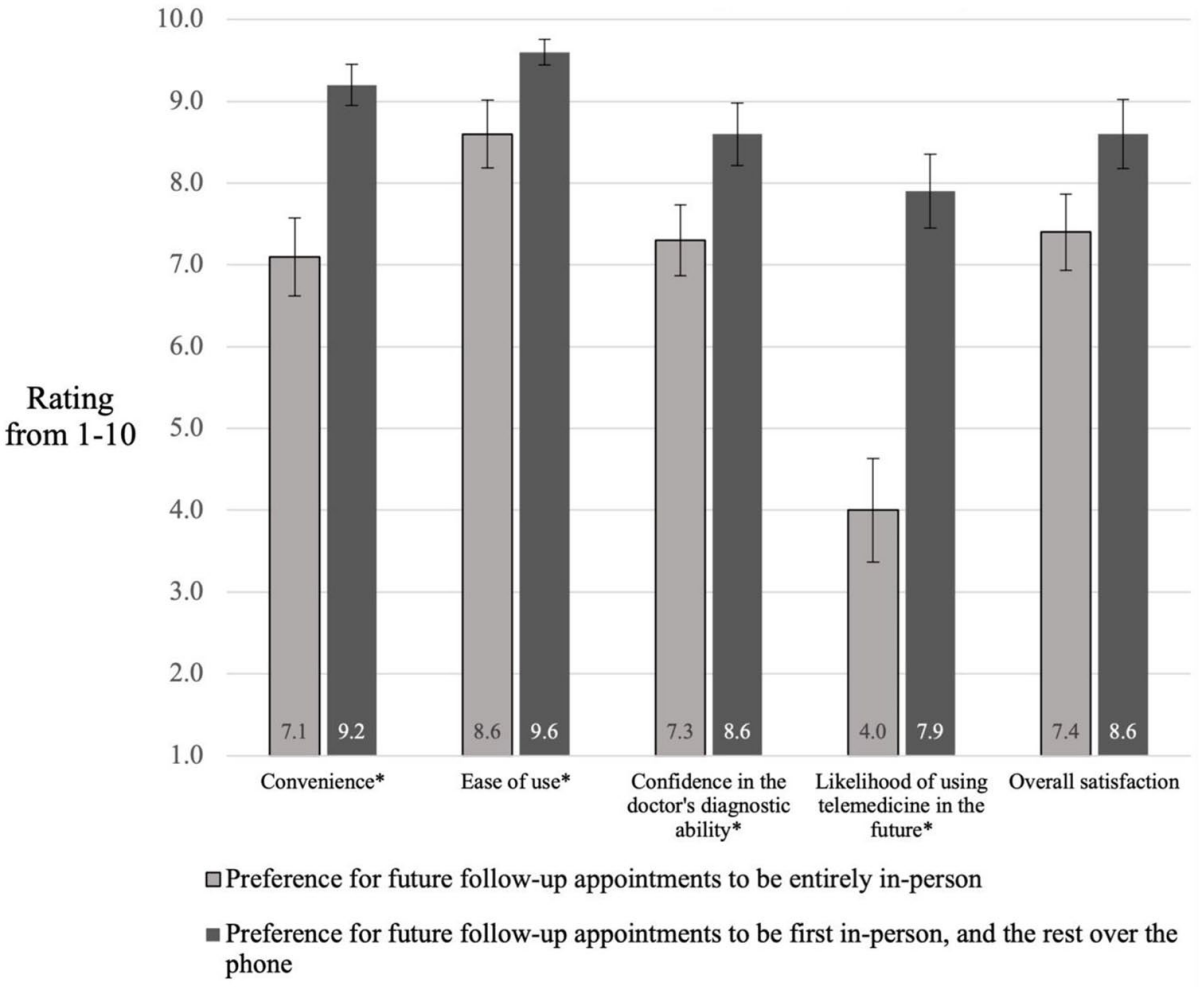
Ratings of various aspects of the telemedicine appointment between groups with different preferences for future follow-up care (*n* = 41). Error bars represent the standard error of the mean. *p < 0.05.

Table 2 represents the participants’ preferred type of appointment if they were to have another surgery in the future. Most notably, a majority preferred to have their first appointment in-person and following appointments over the phone (61.0%). Two-tailed independent samples *t*-tests were used to compare ratings of the telemedicine elements between groups who preferred a blended (telemedicine and in-person) approach for appointments versus those who preferred in-person appointments only. Ratings of telemedicine convenience for the 25 participants who preferred a blended in-person and telemedicine approach (M = 9.2 and SD = 1.2) were significantly higher than those of the 15 who preferred in-person only (M = 7.1 and SD = 1.8) *t*(38) = −4.1, *p* < 0.001 (95% confidence interval (CI) = −3.016 to −1.037). Ratings for the ease of use of telemedicine were significantly higher among those who preferred a blended approach (M = 9.6 and SD = 0.8) than those preferring in-person only appointments (M = 8.6 and SD = 1.6), *t*(17.966) = −2.2, *p* = 0.042 (95% CI = −1.883 to −0.037). Confidence in the doctor’s diagnostic ability via telemedicine was significantly higher in those preferring the blended approach (M = 8.6 and SD = 1.9) compared to the in-person only (M = 7.3 and SD = 1.7), *t*(38) = −2.2, *p* = 0.035 (95% CI = −2.516 to −0.098). The mean rating of the likelihood of using telemedicine in the future among those preferring a blended approach (M = 7.9 and SD = 2.3) compared to those preferring in-person only (M = 4.0 and SD = 2.4) was also significantly higher, *t*(38) = −5.2, *p* < 0.001 (95% CI = −5.459 to −2.381). Ratings of overall satisfaction with the telemedicine appointment between those preferring a blended approach (M = 8.6 and SD = 2.1) compared to in-person only (M = 7.4 and SD = 1.8) showed no significant difference, however, *t*(38) = −1.8, *p* = 0.075 (95% CI = −2.529 to 0.129). These comparisons are demonstrated in Figure 1. There were no significant differences between those who preferred a blended approach versus in-person appointments only with respect to median distance traveled (*U* = 177.5 and *p* = 0.775) nor median time spent in clinic (*U* = 177.5 and *p* = 0.774).

**Table 2.** Preferred method of follow-up care for future surgeries (*n* = 41).

Type of appointment	Frequency of responses (#)	Percentage (%)
In-person only	15	36.6
First in-person, the rest telemedicine	25	61.0
Unsure	1	2.4
Telemedicine only	0	0.0

## Discussion

The COVID-19 pandemic has limited the ability of orthopedic surgeons to conduct in-person examinations in the outpatient setting. With the shift toward telemedicine during the pandemic, this study demonstrates the opportunity costs of attending in-person appointments and also evaluates perceptions of telemedicine among patients. The opportunity costs identified in our results include the travel distance to the clinic, time spent in the clinic, and forgoing typical daily commitments. A similar study by Marsh et al.^11^ found that telemedicine approaches for follow-up surgical care were associated with a reduction in travel costs, travel distance, and average time spent at an appointment compared to a traditional in-person format. In addition, a randomized controlled trial of post-operative telemedicine encounters after rotator cuff repair identified that both in-person and telemedicine groups were highly satisfied with their appointment, but the telemedicine group required less time off work for both themselves and their caregiver during the recovery process.^12^ Given the opportunity costs of attending in-person appointments, telemedicine may be an appropriate alternative for some types of clinic visits.

Our results also demonstrate that patients have a favorable view of telemedicine appointments. Mean ratings of the various elements of the telemedicine appointment showed high scores for convenience, ease of use, confidence in the doctor’s ability to understand or diagnose their condition, and overall satisfaction. The moderate overall score for the likelihood of using the services again is explained by the nearly four point spread when comparing mean scores of participants who would choose blended appointments in the future versus those who chose in-person appointments only. Furthermore, participants who rated the various telemedicine elements higher (except for overall satisfaction, for which there was no significant difference) were more likely to prefer a blended in-person and telemedicine approach for future appointments. These findings are consistent with other studies showing that higher satisfaction scores for video-based appointments were associated with patients choosing that type of appointment again in the future.^13^ Regardless of future preference, a number of studies have found that patient satisfaction is generally high for both in-person and telemedicine appointments.^13–17^

In addition to the high ratings of telemedicine in this study, a majority of patients selected a blended in-person and telemedicine approach for future care if they were to have another surgery. The preference for a blended approach may reflect how patients’ value post-operative reassurance with an in-person visit but also appreciate telemedicine’s convenience over the long term. Furthermore, it may be that telemedicine is preferred when the doctors and patients are already well-known to one another, as suggest by Gilbert et al.^8^ This was reflected in our results, as all telemedicine encounters were follow-up appointments after surgery.

Several similar studies have similarly demonstrated a preference for telemedicine in the future after an initial experience with the service. A study of joint arthroplasty patients who experienced both a web-based telemedicine and an in-person follow-up program found that 44% preferred the telemedicine method, while 36% preferred in-person.^11^ In addition, a randomized controlled trial by Buvik et al.^13^ found that both video-based telemedicine and in-person appointment groups were highly satisfied with their appointment and that 86% of the telemedicine group would prefer the telemedicine appointment in the future. An additional study on pediatric fracture care surveyed patients after their initial telemedicine or in-person appointment on their preferred modality for future care.^17^ Of the 101 patients who initially had their appointment through telemedicine, only eight preferred to have their next appointment in-person.

Despite the similarities of this study to previous work in telemedicine, this study differs because participants could choose a blended version of in-person and telemedicine for future appointments, rather than strictly telemedicine or in-person. In addition, this study used telephone appointments and did not incorporate video services. Researchers in one study identified that patients who had a telephone appointment were more likely to consider using the service again (94% of patients) compared to those who had a video appointment (36% of patients).^8^ This finding suggests that multiple modalities of telemedicine may be seen as acceptable to patients.

Although there have been promising findings on the use of telemedicine in orthopedics, it is important to consider the drawbacks of telemedicine compared to in-person appointments. Relying on smartphones, tablets, or computers may lead to technical difficulties that detract from the clinical visit and introduce inefficiencies in delivering care.^18^ Furthermore, there are medico-legal considerations with telemedicine, such as difficulties performing remote assessments and the responsibility for privacy and confidentiality during virtual appointments.^19^ There are also perceived difficulties with performing physical examinations during telemedicine appointments. Although this may be true for some encounters, several studies have supported that orthopedic surgeons are highly satisfied with virtual physical examinations^12,15,20^ and that measures of physical exam quality^4^ and eventual patient outcomes^21^ are similar between in-person and telemedicine groups. The difficulty of virtual physical examinations in orthopedics is also being mitigated by the publishing of virtual physical examination protocols that may assist physicians in implementing telemedicine into their practice.^22^

There are several limitations to our research study that should be considered in the context of our results. First, the study was conducted during the COVID-19 pandemic, and our results should be considered within this abnormal context. In addition, the study design involves a single center and a single surgeon, thereby reducing both the sample size and sample diversity. Including data from multiple centers and surgeons may yield more generalizable results for different patient populations. In addition, the survey questions were designed to be brief and easily understood over the phone, but patient interpretation may have influenced their responses. For example, the questions about missing work and the associated lost income due to the appointment may have been answered differently between individuals who used paid sick days to attend the appointment. Furthermore, our survey was developed based on previously validated work in telemedicine,^10^ but the modifications that were made to suit the orthopedic practice were not formally validated through a pilot study. Therefore, further evaluation of the validity and test–retest reliability of this survey is warranted.

## Conclusion

The results of this study can be applied to clinical practice and may improve surgical follow-up care. We have first identified that patients undergo a multitude of opportunity costs when attending in-person appointments. In addition, much like the results of previously mentioned studies, we have shown that patients typically have a favorable view toward telemedicine as they see it as a convenient and viable alternative for some appointments. The identified preference for a blended in-person and telemedicine approach for follow-up care suggests that patients appreciate the value of both in-person and telemedicine appointments. Further studies on the use of telemedicine during non-pandemic times may be beneficial for ensuring good patient outcomes.

## Supplemental Material

sj-docx-1-smo-10.1177_20503121211045247 – Supplemental material for Opportunity costs of attending surgical clinic appointments and experiences with telemedicine for follow-up care Click here for additional data file. Supplemental material, sj-docx-1-smo-10.1177_20503121211045247 for Opportunity costs of attending surgical clinic appointments and experiences with telemedicine for follow-up care by Mitchell D Thatcher, Michael W Thatcher, Mckinley C Smith, Michelle McCarron and Jeremy Reed in SAGE Open Medicine
