# Supplementary material for: Opportunity costs of attending surgical clinic appointments and experiences with telemedicine for follow-up care
Source: SAGE Open Med. 2021 Sep 11;9:20503121211045247. doi: 10.1177/20503121211045247 (PMC8436310; doi:10.1177/20503121211045247)
Supplement: sj-docx-1-smo-10.1177_20503121211045247 – Supplemental material for Opportunity costs of attending surgical clinic appointments and experiences with telemedicine for follow-up care [file sj-docx-1-smo-10.1177_20503121211045247.docx]

These first questions are asking about when you normally would come to visit with [blinded] **in-person** at the office:

*Travel:*

1. When you come to [blinded]’s office, what town or city do you usually travel from?

_____________________________________________

1. How much time do you spend travelling one way to the clinic?

_____________________________________________

1. From the time you arrive until the time you leave (including time in the waiting room, with [blinded], etc.) how much time on average do you spend in the clinic?

_____________________________________________

*Effects on Patient:*

1. These next questions you may be answered with yes or no. Because of this appointment, were you unable to participate in any of the following commitments?
   1. ____Work
   2. ____School or education
   3. ____Childcare
   4. ____Recreational activities
   5. ____House or yard work
   6. ____Socializing with friends/family
2. This question assesses the loss of income that patients can face, and you may choose to not answer if you like. Because of this appointment, were you unable to earn income totalling any of the following amounts:

a. ____ Did not miss out on any income

b. ____ $0 to $50

c. ____ $50-$100

d.____ $100-$150

e. ____ $150-$200

f. ____ Over $200

h. ____ prefer not to say

*Telemedicine:*

This portion of the interview is now referring to the appointment that you had with [blinded] **over the phone**. For each of these next questions, I will ask you to provide a score out of 10, with a 10 being the most positive or favourable response, and 1 being the least positive response.

1. _____ How would you rate the convenience of using a phone call appointment?
2. _____ How easy was it to use this service for the first time with [blinded]?
3. _____ How confident are you in the ability of the doctor to understand or diagnose your health concerns over the phone?
4. _____ If you have the choice, how likely are you to use phone call appointment services in the future?
5. _____ Overall, how satisfied are you with your appointment over the phone?

For the final question I will have you choose one of the options that I provide

11. If you were to have another surgery in the future, and the follow-up appointments after you leave the hospital are safe to be done over a phone call, would you prefer to have your appointments:

1. In-person
2. First visit in-person, and following visits over the phone
3. Entirely over the phone
4. Unsure
